# COVID-19 in India: transmission dynamics, epidemiological characteristics, testing, recovery and effect of weather

**DOI:** 10.1017/S0950268820001776

**Published:** 2020-08-11

**Authors:** Arnab Chanda

**Affiliations:** 1 Centre for Biomedical Engineering, Indian Institute of Technology (IIT), Delhi, India; 2 Department of Biomedical Engineering, All India Institute of Medical Sciences (AIIMS), Delhi, India

**Keywords:** Coronavirus, COVID-19, epidemiology, India, transmission, weather

## Abstract

The spread of COVID-19 is recent in India, which has within 4 months caused over 190 000 infections, as of 1 June 2020, despite four stringent lockdowns. With the current rate of the disease transmission in India, which is home to over 1.35 billion people, the infection spread is predicted to be worse than the USA in the upcoming months. To date, there is a major lack of understanding of the transmission dynamics and epidemiological characteristics of the disease in India, inhibiting effective measures to control the pandemic. We collected all the available data of the individual patients, cases and a range of parameters such as population distribution, testing and healthcare facilities, and weather, across all Indian states till May 2020. Numerical analysis was conducted to determine the effect of each parameter on the COVID-19 situation in India. A significant amount of local transmission in India initiated with travellers returning from abroad. Maharashtra, Tamil Nadu and Delhi are currently the top three infected states in India with doubling time of 14.5 days. The average recovery rate across Indian states is 42%, with a mortality rate below 3%. The rest 55% are currently active cases. In total, 88% of the patients experienced symptoms of high fever, 68% suffered from dry cough and 7.1% patients were asymptomatic. In total, 66.8% patients were males, 73% were in the age group of 20–59 years and over 83% recovered in 11–25 days. Approximately 3.4 million people were tested between 1 April and 25 May 2020, out of which 4% were detected COVID-19-positive. Given the current doubling time of infections, several states may face a major shortage of public beds and healthcare facilities soon. Weather has minimal effect on the infection spread in most Indian states. The study results will help policymakers to predict the trends of the disease spread in the upcoming months and devise better control measures.

## Introduction

**Fig. 1.**
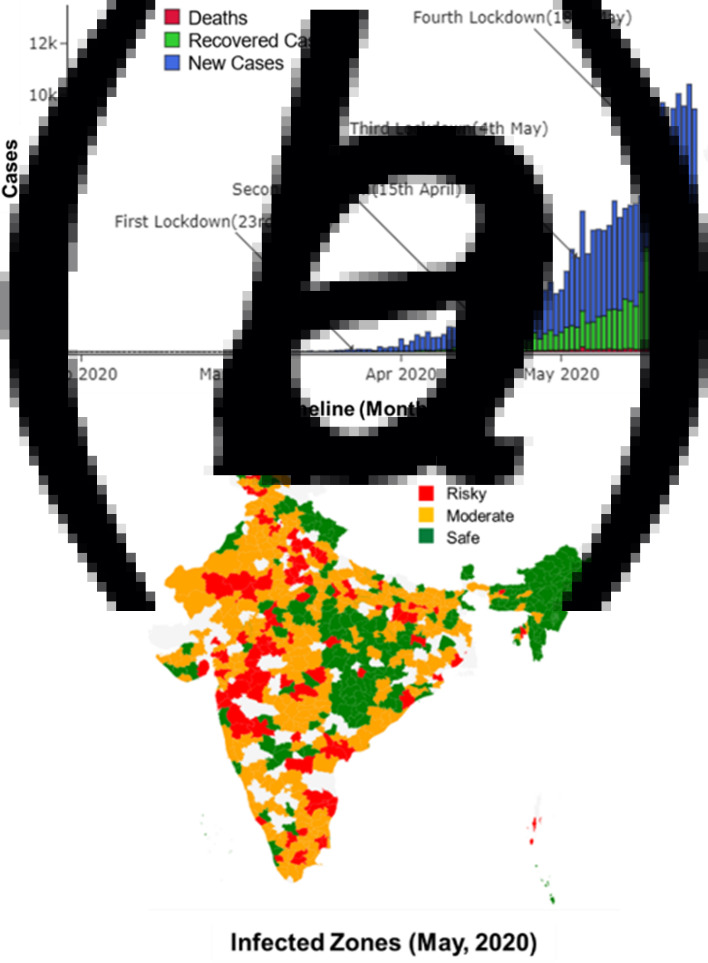
(a) Reported new and recovered cases, and deaths, due to COVID-19 until May 2020 in India. (b) Infected zones declared in India as of May 2020.

The Coronavirus disease (COVID-19) has led to 6.2 million cases of infection, and over 374 000 deaths worldwide, as of 1 June 2020 [1]. With its advent in Wuhan, China, on 31 December 2019 [2], the disease has spread massively in Europe and the USA, affecting over 2.1 and 1.8 million people in these regions, respectively. On 30 January 2020, the first case of COVID-19 was reported in India [3]. As of 1 June 2020, the disease has spread to over 190 000 people and caused 5400 deaths [1]. Within this approximately 4-month timeline, four stringent national lockdowns were implemented (Fig. 1a). Despite such strict measures, the rapid rise of cases in India [4], which is a very densely populated country and home to over 1.35 billion world population, is a grave concern for the upcoming months.

Between 30 January 2020 and 1 April 2020, the Integrated Disease Surveillance Programme (IDSP), which is a decentralised surveillance mechanism in India, was employed. IDSP uses an indicator and event-based surveillance to detect outbreaks early [5]. Due to the disruption of routine health care services during this period, there was low passive reporting of cases. Active surveillance was being done only for those with travel history and in the form of contact tracing of confirmed COVID-19 cases. Heavy testing efforts started around 1 April 2020, when the total reported cases in India increased to approximately 2000. In light of scarce resources, a not-so-robust health care system, and good telecom support with 1.18 billion wireless subscribers in India, the Aarogya Setu app was launched on 2 April 2020. This is a pioneering participatory disease surveillance (PDS) initiative in India [6], supplementing the existing IDSP by finding missing cases and having faster aggregation, analysis of data and prompt response measures. By 13 May 2020, over 100 million installations of this coronavirus tracker app in 11 different languages were reported, and this app was made mandatory for entry and travel purposes across the country. Also, by 25 May 2020, 3 432 006 people had been tested in labs across India, out of which, approximately 4% were reported COVID-19-positive. The Aarogya Setu app uses the phone's location data and Bluetooth to assess the proximity of an infected person by looking through databases created by the government of lab-confirmed and self-reported COVID-19 cases. Besides risk profiling and contact tracing, dynamic information on COVID-19-positive cases in a radius of 500 m–10 km from the user is provided through this app, enabling protection from infection and reducing unnecessary contact with the overburdened health care system. Also, based on the user's gender, age, symptom details, comorbidities, travel and contact history, they are advised on the measures to be taken based on the risk assessment (e.g. isolation, log temperature every 2 h) and for testing, with details of control rooms and testing centres available in their area [6]. Along with such pandemic control strategies at the individual level, the combination of IDSP and PDS surveillance system in India ensures that rapid reporting of confirmed and suspected cases in the community is available for the government and policy makers to reassess the situation timely. Geotagging of cases helps in initiating control measures on-field situations by the authorities (including identification and containment of clusters (Fig. 1b)) and in informing the community of additional precautions needed.

Underreporting has been observed with the deaths of patients with comorbidities [7]. Some examples include cancer patients who are unable to get to the hospital, COVID-19 patients who committed suicide or a migrant worker who died of exhaustion walking home during lockdown. In India, not all deaths are registered and only a small fraction has an identified cause [8]. Also, some political reasons play a role while declaring deaths. In several regions, lower deaths were reported intentionally to not lose public trust on the local political party. These factors considerably increase the likelihood of missing COVID-19 deaths. Compared to 4–8% deaths reported in developed countries such as the USA, Europe and Japan, less than 3% deaths were reported in India and other developing countries such as Russia.

Tiwari *et al*. [9] have predicted the transmission in India through modelling of cases in China using machine learning, and emphasised on implementation of aggressive lockdowns to control the spread. Lockdowns have been imposed in a majority of countries, over the past few months, having a significant number of active COVID-19 cases [10]. While lockdowns have been claimed as a way to implement effective social distancing and subsequently flatten the curve [11], its benefits have not been observed much in India [12]. The first lockdown across all Indian states was imposed on 23 March 2020, when the total reported cases were just 499 [1]. The second lockdown was implemented on 15 April 2020 in view of the increasing cases (over 1000/day) and total reported cases above 10 000. On 4 May 2020, the total cases went over 50 000, and the third lockdown was imposed [1]. However, by this time, the country was facing massive economic and hunger crisis due to the unemployment of the daily wagers [13]. To assuage such losses, the Indian government provided several relaxations, including the resumption of flights, public transport, and also offices and factories in non-contaminated (green) zones [14] (Fig. 1b). By 18 May 2020, the total reported cases increased to 100 000 with approximately 4500 daily rate of increase. The fourth lockdown was forced on this date as an extension of the third lockdown, which has not had much success as of 1 June 2020 as the cases tend to rise rapidly [1]. Ambikapathy and Krishnamurthy [15] had already predicted similar trends, where any relaxation in lockdown measures was anticipated to cause an exponential rise in transmissions. Such failures in containing the rapidly spreading virus through lockdowns indicate a lack of understanding of the transmission dynamics of the disease in India and other possible factors which may be supporting the spread, warranting an immediate investigation.

In this work, we studied the initial causes and spread of COVID-19 infections in India through an investigation of case reports. Along with the study of the common symptoms, the recovery and death trends were also investigated. A comprehensive analysis was conducted to understand the effect of age, gender, common symptoms, population, testing rate, healthcare facilities and also weather, on the disease spread in India. Our findings will not only be valuable for policymakers in India to better control the disease spread, but also provide indispensable information for epidemiologists and scientists to effectively plan outbreak control measures in other affected countries.

## Methods

### New and recovered cases, and deaths

The data on the number of new and recovered cases, and deaths in India were collected daily from the Centre for Systems Science and Engineering (CSSE) repository, John Hopkins University [16] from 1 January 2020 to 25 May 2020.

### Patient information

The data on patients (numbered as per the disease chronology) such as their gender, age, symptoms and treatment outcomes were provided by the Ministry of Health & Family Welfare [17]. This is the most up-to-date and accurate data on COVID-19 cases which have been made available to the public by the government of India. It should be mentioned that no ethical approval was needed or obtained for this study as the datasets we have used have been made openly available to the public (Source: https://www.mohfw.gov.in/), and do not include any patient identifying information.

### Diagnosis and treatment

State-wise information on testing rates and availability of healthcare facilities (i.e. testing labs and hospital beds) was provided by the Ministry of Health & Family Welfare [17].

### Census and weather data

India's Census data including state-wise population and population density were provided by the Ministry of Health & Family Welfare [17]. Weather data such as relative humidity in % and temperature in ^o^C were collected from January 2020 to December 2020, on a daily basis, for the entire world and all Indian states, using the database of Virtual Crossing, Germany [18]. Clausius Clapeyron equation described in [19] was used to estimate absolute humidity or AH (g/m^3^) from temperature and relative humidity data.

### Modelling and data analysis

Data of the first 530 patients were analysed to map the initial transmission dynamics. These patients were categorised into two groups. The first group included the patients who were infected outside of India and transmitted COVID-19 to the second group through contacts during co-travelling or after arrival in India. The second group was further categorised into two subgroups based on if they transmitted infection to only one or more than one person. The travel history of the first group was statistically analysed to determine the percentage distribution of infected cases arriving from different countries into India. The distribution of the second group across all Indian states was also quantified. Additionally, the percentage of cases which occurred due to inter-state travel of the second group was analysed. It should be mentioned that this mapping of transmission was conducted using the reported information, and not through mathematical modelling.

The daily new cases were studied state-wise to understand the local transmission and doubling rates. The recoveries and death trends (i.e. daily occurrences and rates) were also analysed to estimate the outcome of the disease transmission so far in India. The individual patient data of 136 204 cases reported up to 25 May 2020 were analysed using statistical distribution modelling to determine the common symptoms, gender and age distribution, and also the time of recovery. Specifically, 14 symptoms (e.g. fever and dry cough) were plotted against the number of cases which suffered one or more of such symptoms. The number of males and females was counted out of the reported cases. The cases were further distributed across age intervals (i.e. 0–9, 10–19, 20–29, 30–39, 40–49, 50–59, 60–69 and 70–79) in years and time of recovery intervals (1–5, 6–10, 11–15, 16–20, 21–25 and over 25) in days.

The relationship of state-wise cases with population, population density in both rural and urban areas was determined using linear regression modelling. Also, the state-wise testing efforts were tracked during the months of April and May 2020, and the correlation (i.e. *r*^2^) of state-wise positive cases with the state-wise testing was quantified. Additionally, the available number of public beds and healthcare facilities in each state was compared with the number of active cases till 25 May 2020 to understand the upcoming risks. All correlations were separately computed between state-wise cases and the affecting parameter (i.e. population, population density, testing, public beds and healthcare facilities) with a significance level (*α*) of 0.05. It should be mentioned that the interactions between the parameters were not studied.

The relationship of new cases and weather parameters was studied worldwide and the findings were projected on Indian states to determine the possible weather-based spread. For each 10-day block during 21 January–29 April 2020, the total number of new cases and mean absolute humidity (AH) (which is a cumulative measure of temperature (^o^C), relative humidity (%) and have been observed to be a good metric for studying COVID-19 transmission [20]) were estimated across all countries around the world. The cases were then distributed statistically across 12 different three-point AH range intervals (e.g. 3–5 g/m^3^). From the distribution, the vulnerable (or risky) AH ranges with the maximum number of cases were identified. The monthly mean AH across different Indian states were compared with the vulnerable AH ranges during January and December 2020 to understand the risk of weather-based COVID-19 transmission.

## Results

### Initial transmission dynamics

Travel from China and highly affected countries such as Italy and Dubai were the key factors for COVID-19 incidence in India. This was followed with local transmission caused through unknowing personal contacts such as with co-travellers, family members and relatives, manpower (i.e. maids and support staff), at places of leisure and religious gatherings, and interaction with patients assumed to suffer from normal seasonal flu.

**Fig. 2.**
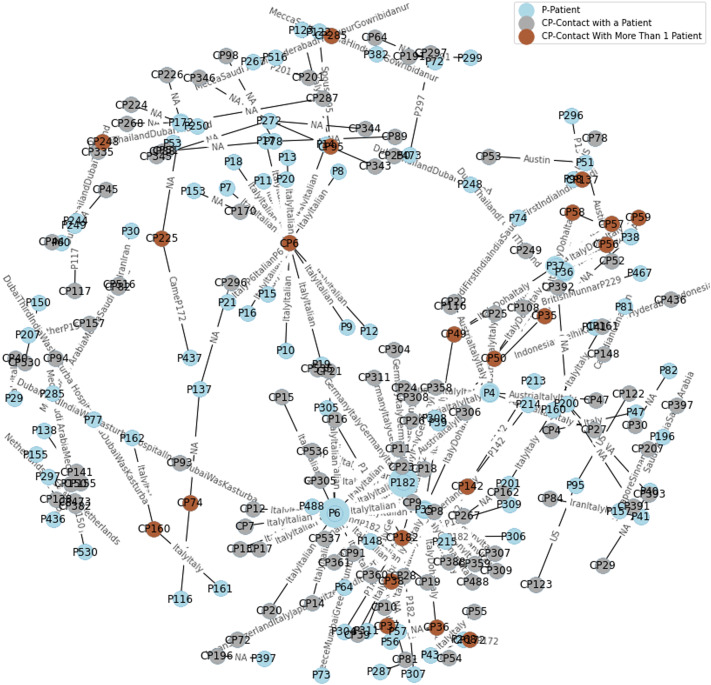
Representation of the first few COVID-19 patients in India, and further disease transmission dynamics across patients who came in direct or indirect contact.

Figure 2 shows a detailed map of some of the significant local transmissions from the initial infected patients (i.e. first group) arriving into India via international flights, to one and more than one new patients (i.e. second group). The first three patients in India arrived from Wuhan, China, the epicentre of COVID-19 outbreak, into Kerala. They were carefully isolated and were declared fully recovered without any further transmission. Out of the first 530 patients with all known details, 6.8% of them travelled from Dubai, 1.79% from the UK and 1.58% from Italy, and initiated local transmission. A significant amount of local transmission (i.e. 59.3%) occurred with patients who travelled to Delhi and came in contact with the initial patients. Only 1.58% who travelled out of Delhi were infected. The other 28.9% had no international or interstate travel history, and were affected via contact with earlier patients.

Overall, approximately 10% were first group patients who were infected out of India, and rest of the patients were infected due to local transmission.

In Maharashtra, which is currently the worst affected state in India, 101 (i.e. 19%) out of first 530 patients with known details were located. To illustrate how complex the transmission was (Fig. 2), we tracked the first case in Maharashtra, which was patient 43 (i.e. first group), who travelled from Dubai with his wife (patient 44). Patient 59 was the daughter of patients 43 and 44. Patient 61 was the cab driver who ferried the family from the airport to home, and patient 60 was a co-passenger. Patients 65–69 were co-passengers with the family on the flight from Dubai.

Out of the 530 patients with known details, the most significant local transmissions were caused in Karnataka by patients 419 and 221, with previous travel history in Dubai and UK, respectively. While patient 419 contributed to 1.79% local transmission, the spread caused by patient 221 was 1.3%.

### Local transmission, recoveries and deaths

As of 25 May 2020, approximately 136 204 cases were reported in India. Out of these cases, 63.4% had either international or interstate travel histories. Across the states, Maharashtra had the maximum number of cases, followed by Tamil Nadu, Gujrat and Delhi (Table 1). The doubling time of the infections was estimated for the 10 Indian states with the most cases. Maharashtra with the maximum number of cases had a doubling time of 14.50 days, similar to Tamil Nadu and Delhi. Gujrat and Madhya Pradesh exhibited an infection doubling time of approximately 24.31 days. For Rajasthan, Uttar Pradesh, West Bengal and Bihar, the doubling times were approximately 18.25, 16.8, 17.4 and 12.8 days, respectively. Also, currently, 55% of the reported cases are active, and are being treated in hospitals across India.

**Table 1.** State-wise distribution of total cases, recoveries and deaths in India till 25 May 2020

State	Total cases	Deaths	Recovered	Recovery rate	Death rate
Maharashtra	50 231	1635	14 600	0.29	0.03
Tamil Nadu	16 277	111	8324	0.51	0.01
Gujarat	14 056	858	6412	0.46	0.06
Delhi	13 418	261	6540	0.49	0.02
Rajasthan	7028	163	3848	0.55	0.02
Madhya Pradesh	6665	290	3408	0.51	0.04
Uttar Pradesh	6268	161	3538	0.56	0.03
West Bengal	3667	272	1339	0.37	0.07
Andhra Pradesh	2823	56	1856	0.66	0.02
Bihar	2587	13	702	0.27	0.01
Karnataka	2089	42	654	0.31	0.02
Punjab	2060	40	1898	0.92	0.02
Telangana	1854	53	1090	0.59	0.03
Jammu & Kashmir	1621	21	809	0.5	0.01
Odisha	1336	7	550	0.41	0.01
Haryana	1184	16	765	0.65	0.01
Kerala	847	4	521	0.62	0
Assam	378	4	55	0.15	0.01
Jharkhand	370	4	148	0.4	0.01
Uttarakhand	317	3	58	0.18	0.01
Chhattisgarh	252	0	67	0.27	0
Chandigarh	238	3	186	0.78	0.01
Himachal Pradesh	203	3	63	0.31	0.01
Tripura	191	0	165	0.86	0
Goa	66	0	19	0.29	0
Ladakh	52	0	43	0.83	0
Puducherry	41	1	12	0.29	0.02
Andaman and Nicobar Islands	33	0	33	1	0
Manipur	32	0	4	0.12	0
Meghalaya	14	1	12	0.86	0.07
Dadar Nagar Haveli	2	0	0	0	0
Nagaland	1	0	0	0	0
Sikkim	1	0	0	0	0
Mizoram	1	0	1	1	0
Arunachal Pradesh	1	0	1	1	0

Also estimated are the state-wise recovery and death rates.

The distribution of recovered cases across Indian states followed the same order as the total cases. The total and recovered cases exhibited a high degree of correlation (*r*^2^ = 0.92, *P* < 0.001, *t* = 20.09). The maximum recovery rate of 0.92 was observed in Punjab, where 1898 patients were already recovered out of 2060 cases. Meghalaya and Tripura shared the second largest recovery rate of 0.86. The third highest recovery rate (i.e. 0.83) was in Ladakh. It should be noted though that the total number of cases in either of Meghalaya, Tripura and Ladakh was below 200, which represented a negligible percentage of total cases reported in India. The minimum recovery rates (<0.20) were observed in Manipur, Assam and Uttarakhand, with the maximum number of cases reported in any of these states below 400. Among the states with the maximum number of cases, Maharashtra was estimated to have a recovery rate of 0.29, followed by Tamil Nadu with 0.51, Gujrat with 0.46 and Delhi with 0.49 rates of recoveries. The average recovery rate for India was estimated to be 0.42 (i.e. 42%).

Deaths were the highest in Maharashtra, followed by Gujrat, Madhya Pradesh, West Bengal and Delhi. The correlation of the total cases with deaths (*r*^2^ = 0.87, *P* < 0.001, *t* = 15.18) was lower than that with the recoveries. The correlation between recoveries and deaths was low (*r*^2^ = 0.75, *P* < 0.001, *t* = 10.21). The maximum state-wise death rate was 0.07, in Meghalaya with 14 total cases and West Bengal with 3667 total cases. This was followed by Gujrat with a 0.06 death rate among 14 056 total cases. In the most affected Maharashtra, Delhi, Rajasthan, Madhya Pradesh and Uttar Pradesh with over 5000 total cases, the death rates were below 0.5. For all other states, the death rates were in the range of 0.01–0.03. The national death rate was estimated to be below 0.03 or 3%.

### Clinical characteristics of infected patients

**Fig. 3.**
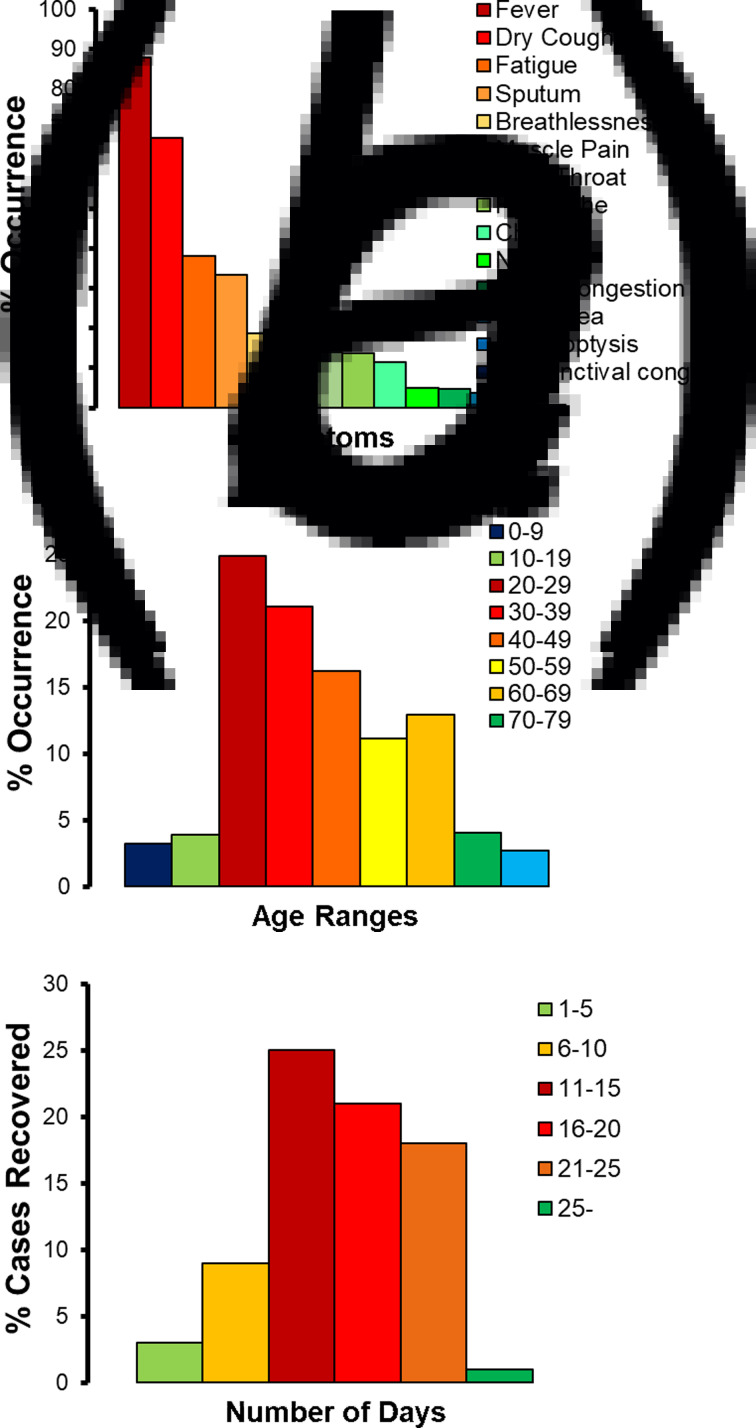
COVID-19 patients in India till 25 May 2020: (a) percentage (%) occurrence of different symptoms in, (b) percentage (%) occurrence across different age ranges, (c) percentage (%) cases recovered within different number of days.

The common symptoms were studied across the 136 204 cases reported in India till 25 May 2020. In total, 88% of the patients suffered from high fever and 67.7% complained of dry cough (Fig. 3a). In total, 38.1% patients also suffered fatigue, and 14.8% developed muscle pain. In total, 33.4% patients experienced sputum production and thick mucus coughed up from the lungs. Breathlessness was also observed in 18.6% of the patients. Sore throat, headache and chills were also reported in 11–14% of the patients. Nausea and nasal congestion were experienced by approximately 5% patients. A less common symptom of diarrhoea complemented with other symptoms in just 3.7% patients. A very small section of the patients (i.e. less than 1%) also reported the symptoms of haemoptysis (i.e. coughing up blood), and few more complained of conjunctival congestion. Approximately 7.1% patients who tested COVID-19-positive did not have any symptoms at all (i.e. asymptomatic).

Based on the study of the gender distribution across the 136 204 cases reported in India till 25 May 2020, approximately 66.8% were identified to be males. Age distribution modelling (Fig. 3b) identified over 73.3% cases in India to have been reported in adult patients who were in the age group of 20–59 years. The most vulnerable age range was 20–29 years, representing approximately 24.9% of the patients, followed by the age range of 30–39 years (with 21.1% patients) and 40–49 years (16.2% patients). The patients in the age group of 50–59 years represented 11.1% of the COVID-19-positive cases. In total, 19.6% of the retired and senior citizens above 60 years of age were infected, with a skew towards the age group of 60–69 (with 12.9% patients). The children and teenagers of ages 0–9 and 10–19 years represented 3.2% and 3.9% of the patients, respectively.

The time taken for complete recovery of the COVID-19 patients was tracked (Fig. 3c). Over 83% of the Indian patients recovered in 11–25 days. Out of this pool, maximum number of recoveries (i.e. 32.4%) happened in 11–15 days and 27.2% recoveries took place in 16–20 days. In total, 23.3% patients took 21–25 days to recover fully. Approximately, 11.7% patients with mild symptoms recovered in 6–10 days. Only 3.9% of the patients recovered in 1–5 days. Very few patients (i.e. 1.3%) took over 25 days to recover.

### Effect of population and population density

**Fig. 4.**
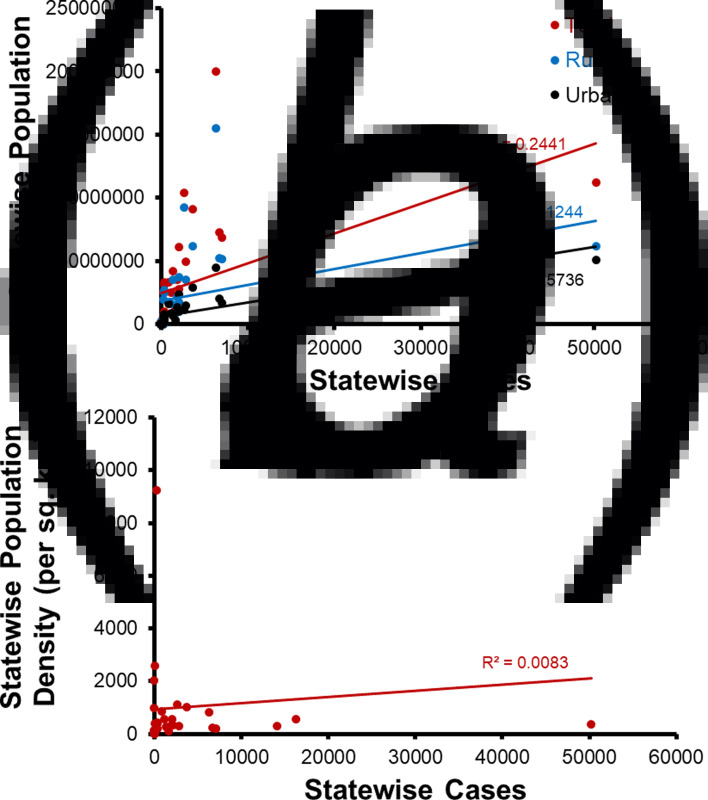
State-wise COVID-19 cases in India *vs*. (a) state-wise total, urban and rural population, (b) state-wise population density.

The correlation between the COVID-19 cases and population was estimated (Fig. 4). State-wise total population is poorly correlated (*r*^2^ = 0.24, *P* < 0.05, *t* = 3.3) with state-wise total cases reported till 25 May 2020. The state-wise rural population has almost no correlation (*r*^2^ = 0.12, *P* < 0.05, *t* = 2.1) with state-wise total cases. However, the state-wise urban population exhibited a moderately high correlation (*r*^2^ = 0.57, *P* < 0.001, *t* = 6.7) with state-wise total cases. Furthermore, state-wise population density had no correlation (*r*^2^ = 0.008, *P* = 0.59, *t* = 0.53) with state-wise total cases.

### Effect of testing rate and healthcare infrastructure

**Fig. 5.**
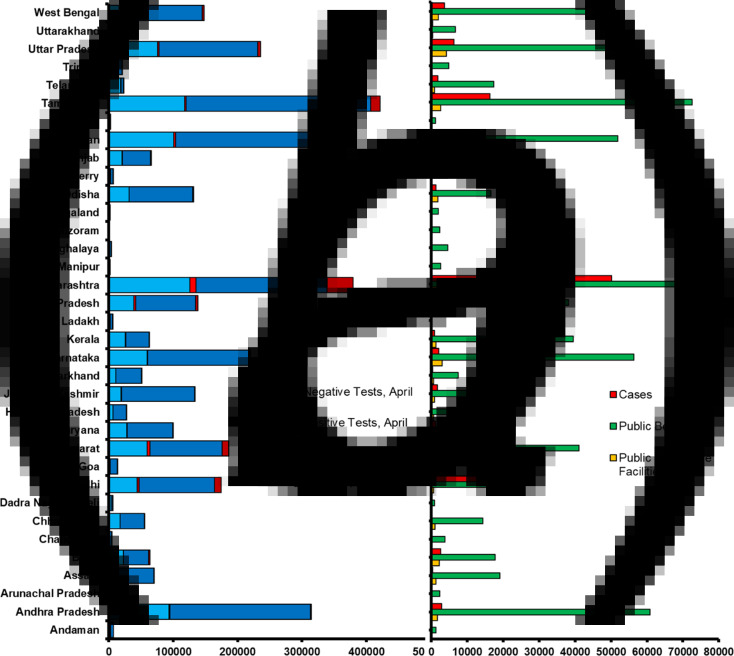
State-wise distribution of: (a) positive and negative tests in April and May 2020, (b) total cases, available public beds and healthcare facilities till May 2020.

Very few people were tested for COVID-19 in India till the end of March 2020. Heavy testing efforts started around 1 April 2020 when the total reported cases increased to approximately 2000. Figure 5a shows a distribution of the state-wise test outcomes (i.e. positive or negative) in April and May 2020. By 25 May 2020, 3 432 006 people were tested in India. Out of these, approximately 4% were detected COVID-19-positive. Across the states, Tamil Nadu conducted 421 450 tests in total, followed by Maharashtra, where 397 185 tests were completed. Also, over 100 000 tests were conducted separately in Rajasthan, Andhra Pradesh, Uttar Pradesh, Karnataka, Gujrat, Delhi, West Bengal, Madhya Pradesh, Jammu and Kashmir and Odisha. The lowest number of tests was conducted in Mizoram, Nagaland, Manipur, Sikkim, Meghalaya, Chandigarh, Ladakh, Dadra Nagar Haveli, Arunachal Pradesh, Puducherry and Andaman. The mean of percentage tests conducted in all states with respect to their population was estimated to be less than 0.5%. Also, the correlation between the state-wise total number of tests and positive cases was moderate (*r*^2^ = 0.48, *P* < 0.001, *t* = 5.5).

The available number of public beds in different states of India currently exceeds the number of COVID-19 patients (Fig. 5b). The maximum number of public beds is available in Tamil Nadu followed by Maharashtra. Also, Andhra Pradesh, Karnataka, Rajasthan, Uttar Pradesh and West Bengal have a significant number of beds (i.e. over 50 000). Below 5000 public beds are only available in Andaman, Arunachal Pradesh, Chandigarh, Dadra Nagar Haveli, Goa, Lakshadweep, Manipur, Mizoram, Nagaland, Sikkim, Tripura, Puducherry and Meghalaya. If the cases rise in these states, it will be difficult to manage the situation with such a low number of beds. To date, over 50% of public beds are occupied in Maharashtra and Delhi, which are at a risky juncture. Once the cases double in these states in the coming days, the available beds will not be enough for treatment. Other states with above 10% occupancy of public beds and some future risk include Bihar, Gujrat, Jammu and Kashmir, Madhya Pradesh, Punjab, Rajasthan, Tamil Nadu, Telangana and Uttar Pradesh. The overall correlation of state-wise number of cases and available beds is *r*^2^ = 0.38, *P* < 0.001, *t* = 4.48.

### Effect of weather

**Fig. 6.**
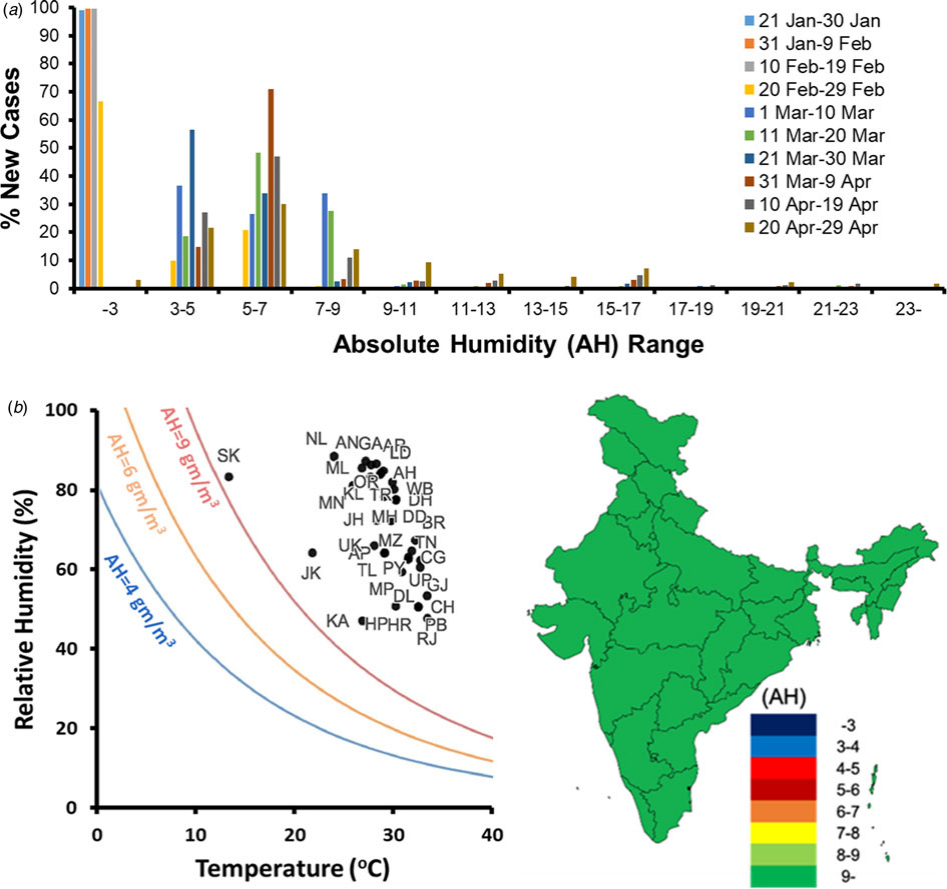
(a) Distribution of new cases worldwide across absolute humidity (AH) ranges in different 10-day blocks between January and April 2020. (b) AH across Indian states in June 2020.

The relationship of infected cases with weather parameters such as temperature (T), relative humidity (RH) and absolute humidity (AH) was modelled during January–April 2020 across all countries around the world. While temperature was found to not be correlated with the disease spread at all, a risky absolute humidity range of 3–9 g/m^3^ was identified, in which, over 90% of infected cases were reported after the initial incidence in absolute humidity below 3 g/m^3^ (Fig. 6a). These findings projected on India during different months in 2020 indicated that a majority of the states in India will fall outside the risky absolute humidity ranges throughout the year [20]. During January–May 2020, Sikkim, Himachal Pradesh, Rajasthan, Jammu and Kashmir, Chandigarh, Haryana and Gujrat were the only states which in some weeks experienced the risky absolute humidity range of 3–9 g/m^3^. From June to September 2020 which are also the monsoon months in India, none of the states will be in the risky weather range. Figure 6b shows a glimpse of the expected weather conditions in different states of India during the month of June 2020. Between October and December 2020, Jammu and Kashmir, and Sikkim are expected to fall in the risky absolute humidity range again. It should be mentioned that there is no existing evidence to date of weather causing the onset of COVID-19 spread in any Indian state. The predictions of weather supporting the spread in the upcoming months are mere correlations and projections based on worldwide observations.

## Discussion

This study generated novel findings on the transmission dynamics, epidemiological and clinical characteristics of COVID-19 in India. While a majority of initial transmission could be attributed to the co-travelling with patients from abroad into different states, significant local transmission occurred through a complex chain of interactions with the family and during local travel. Along with the rapid spread of the disease so far across Indian states, the recoveries have been high and deaths have been low. Most patients in India experienced high fever and dry cough symptoms during the initial onset of the infection, and were males in the age range of 20–59 years. In average, most patients recovered within 11–25 days. The spread was also observed to be more in the urban population compared to in rural population. While massive testing was conducted across all Indian states between 1 April and 25 May 2020, many of the highly affected states are likely to face shortage of public beds and healthcare infrastructure within the next few months. The upcoming monsoons and colder months are not expected to affect the spread of COVID-19 much in 2020.

Many of our findings are very similar to worldwide observations. The initial transmission via air travel and subsequent local transmission to family members and acquaintances, as observed in Indian population, is a common occurrence in other countries such as the USA and Italy [21]. The below 3% mortality rate observed in India is almost half of the average worldwide mortality rate of 6%, but similar to other countries such as Turkey [1]. The most common symptom of fever followed by dry cough, and gender bias, reported in Indian patients are also very similar to that observed in other patients worldwide [22]. Additionally, the disease spread has been predicted to be least affected by weather in tropical climate of India, consistent with other studies [20].

Social distancing along with the use of personal protective equipment (PPE) may reduce local transmissions during travel. In case of any symptoms of fever or dry cough, immediate self-quarantine for at least over 25 days may prevent other family members from getting infected. The containment zones in the upcoming phases of lockdown should follow similar protocols. Given the high recovery rates in India, seeking timely medical attention is important to avoid deaths. In the upcoming monsoon, people developing seasonal flu conditions may suspect COVID-19 infection out of fear and panic, leading to an unprecedented burden on the healthcare system. The state-wise number of testing and hospital beds has to be ramped up immediately in light of these predictions, and with the cases rising rapidly in India. If the disease spread continues beyond October 2020, a second wave of infection is expected supported by weather.

There are a few limitations of this study. The alternative modes of local transmission, including airborne and surface-based spread, were not characterised across patients. The effect of pre-existing health conditions (e.g. hypertension and diabetes [23]) on the incidence and severity of COVID-19 infection was not considered in the clinical characterisation of the Indian patients. As cases have been rising in India, besides increasing public hospital beds, several institutions and unutilised public spaces are being converted into quarantine facilities. The number of cases, such new units can handle, was not considered while estimating the state-wise burden on healthcare infrastructure in the upcoming months. In the monsoon months (June–September 2020), while weather may not support the spread, the role of comorbidity caused by common flu or mosquito-borne diseases (i.e. dengue) was not considered. With the availability of more detailed datasets, such factors will be incorporated into future studies to fully understand the transmission dynamics of the disease in India. Also, it should be mentioned that some of the results are specific to the Indian population, and caution should be utilised when extrapolating them to other regions.

## Conclusion

The study for the first time investigated the initial and local transmission of COVID-19 in India. The common symptoms, age, gender and recovery time of the patients were determined statistically. The state-wise distribution and growth of cases, recovery rates and deaths were studied. The effect of population, testing, healthcare infrastructure and weather on the disease spread was also analysed. This information can lead to a better understanding of the transmission dynamics of the disease in India. The results provide important guidelines for diagnosis, prevention and controlling of the disease in the upcoming months.

## Data Availability

The data on cases, recoveries and deaths in India are available from the Centre for Systems Science and Engineering (CSSE) repository, John Hopkins University (https://coronavirus.jhu.edu/). The data on Indian patients, state-wise testing and healthcare facilities, and population are available from the Ministry of Health & Family Welfare (https://www.mohfw.gov.in/). Worldwide weather data are available through Virtual Crossing Database, Germany (https://www.visualcrossing.com/weather-api).
