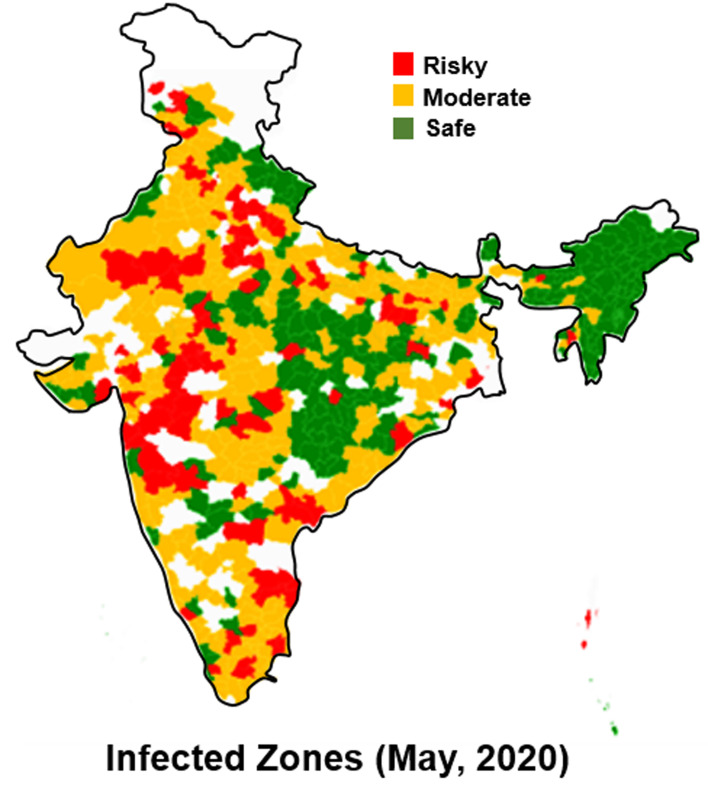# COVID-19 in India: transmission dynamics, epidemiological characteristics, testing, recovery and effect of weather – Corrigendum

**DOI:** 10.1017/S0950268820002411

**Published:** 2020-10-23

**Authors:** Arnab Chanda

**Affiliations:** 1 Centre for Biomedical Engineering, Indian Institute of Technology (IIT), Delhi, India; 2 Department of Biomedical Engineering, All India Institute of Medical Sciences (AIIMS), Delhi, India

It has been brought to our attention that Figure 1B published in ‘COVID-19 in India: transmission dynamics, epidemiological characteristics, testing, recovery and effect of weather’ showed an incorrect depiction of India’s international boundary.

The figure has been corrected as follows:

**Figure:**